# Supplementary material for: Patterns of Intron Gain and Loss in Fungi
Source: PLoS Biol. 2004 Nov 30;2(12):e422. doi: 10.1371/journal.pbio.0020422 (PMC532390; doi:10.1371/journal.pbio.0020422)
Supplement: Table S1 — Also available at http://genes.mit.edu/NielsenEtAl/. (4.3 MB ZIP). [file pbio.0020422.st001.zip › NielsenEtAl/html/518.html]

AN5577.1.NCU09560.1.MG00212.1.FG04454.1


```
 CLUSTAL W (1.82) Multiple Sequence Alignments - Introns Inserted


Sequence 1: MG00212.1	227 aa
Sequence 2: FG04454.1	230 aa
Sequence 3: NCU09560.1	228 aa
Sequence 4: AN5577.1	223 aa
Alignment Length: 232 aa
Number Identitical Residues: 127 aa
Alignment Score (without introns) 5952


MG00212.1 	MAS-LIRTTPAVRGALRSRAFKPAAMASTSFVRGKATLPDLKY1DYGALEPYISAEIMEL
NCU09560.1	MSASLFRATPVARSALRAAS---AVKPAATFVRGKATLPDLQY1DYGALEPYISSKIMEL
FG04454.1 	MASALLRSTPALRAGLRARSTPLAAMATTSFVRGKATLPDLPY1DYGALEPYISGQIMEL
AN5577.1  	MAS-LIRTS--LRTGLRASS---SSAAPLTFTRGKATLPDLAY1DYGALEPAISGKIMEL
          	*:: *:*::   * .**: :   :  .. :*.********* * ******* **.:****

MG00212.1 	HHSKHHNTYVQGYNSAVQAIAEAPTP---QAAAAVAPLLNFHGGGHVNHSLFWENLAPAS
NCU09560.1	HHSKHHQTYVNGLNSALTTIAEAESKGDFTKAASVAPLLNFHGGGHLNHTLFWENLAPAS
FG04454.1 	HHSKHHQTYVTGFNNATDAIAEANHKGDAKAAAAQAPLLNFHGGGHVNHSLFWENLAPNG
AN5577.1  	HHKNHHQTYVNSYNTAIEQLQEAQASNNIAAQIALKPLINFHGGGHINHTLFWENLAPKN
          	**.:**:*** . *.*   : **   ..     :  **:*******:**:******** .

MG00212.1 	REGGGEPDGKLRAGIDATFGSFETFTKQMNTTLAGIQGSGWAWLAKDKTT-GALSIVTRA
NCU09560.1	REGGGSPDGALGKAITTEFGSFESFVKQMNAALAGIQGSGWAWLAKDKAANGKLALITRA
FG04454.1 	KGGGGEPEGKLLTSINEDFGSFEAFKKQTNATLAGIQGSGWAWLVKDKNA-GTLSIVTRP
AN5577.1  	AGGGEPPSGALSKAINESFGSLENFQGQMNTALAAIQGSGWAWLVQDKQT-GSIAIKTYA
          	  **  *.* *  .*   ***:* *  * *::**.*********.:** : * ::: * .

MG00212.1 	~NQDPVAGNLAPLLGIDAWEHAYY~LQYQNRKAEYFSAIWNVINWKTVGNRFDKS
NCU09560.1	~NQDPVTGNYVPLLGIDAWEHAYY~LQYENRKAEYFEAIWNVINWKTVAKRFEA-
FG04454.1 	~NQDPVTGNLEPLLGIDAWEHAYY~LQYQNRKAEYFSAIWDVINWGTVAKRFEK-
AN5577.1  	0NQDPVVGQFKPLLGIDAWEHAYY2LQYQNRKAEYFKAIWEVINWKAVEKRFA--
          	 *****.*:  ************* ***:*******.***:**** :* :**
```
